# Genomic landscape of inflammatory breast cancer identifies potential actionable genetic alterations

**DOI:** 10.18632/oncoscience.515

**Published:** 2020-06-30

**Authors:** François Bertucci, Steven Van Laere, Daniel Birnbaum

**Affiliations:** ^1^Laboratoire d’Oncologie Prédictive, Centre de Recherche en Cancérologie de Marseille (CRCM), Inserm, U1068, CNRS UMR7258, Institut Paoli-Calmettes, Aix-Marseille Université, Marseille F-13009, France; ^2^Département d’Oncologie Médicale, Institut Paoli-Calmettes, Marseille, France; ^3^Department of Oncological Research, GZA Hospitals Sint-Augustinus, Antwerp, Belgium

**Keywords:** DNA repair, Inflammatory breast cancer, Next-Generation Sequencing, NOTCH

Inflammatory breast cancer (IBC) is the most aggressive clinical form of breast cancer. Despite therapeutic progresses, ~50% of patients die from metastatic relapse. Since the last two decades, efforts have been made to better characterize IBC molecular biology and identify new therapeutic targets. High-throughput molecular analyses have been applied to clinical samples [1], mainly based on gene expression profiling [2]. But IBC remains insufficiently characterized because of the scarcity of the disease, the small size of diagnostic samples, and the heterogeneity of studies with respect to the composition of the IBC and non-IBC control groups. The four pioneering studies based on Next-Generation Sequencing (NGS) [3-6] concerned small series (<54 IBCs), including both untreated primaries (<26 cases) and pre-treated relapses. The number of tested genes varied between 50 to 255 in the three series studied by targeted NGS [3-5]. Few studies directly compared primary IBC and non-IBC, and the comparisons were not adjusted upon the molecular subtypes despite the imbalance between IBC and non-IBC and were not corrected for multiple tests. Two of the most recurrently mutated genes identified in IBC (*TP53, HER2/ERBB2*) were associated with molecular subtypes (i.e. triple-negative, HER2+, respectively). The main finding was an increased tumor mutational burden (TMB) in IBC translating in the presence of many actionable genetic alterations (AGAs) with low frequency. Recently, a larger and more homogeneous series was reported [7]. In a molecular subtype-adjusted analysis of 91 genes in non-pretreated primary tumors of 156 IBCs and 197 stage 3-4 non-IBCs, 17 genes were more frequently mutated in IBC, including – in decreasing order of frequency in IBC - *TP53, NOTCH2, MYH9, BRCA2, ERBB4, POLE, FGFR3, ROS1, NOTCH4, LAMA2, EGFR, BRCA1, TP53BP1, ESR1, THBS1, CASP8, and NOTCH1*. The analysis was not corrected for multiple tests.

Based on these observations, we launched a large multicentric comparative NGS study of non-pretreated primaries from IBC and non-IBC patients treated in our institutions, pooled with publicly available NGS data [8]. IBC was clinically defined as T4d according to the consensus criteria. The whole series included 101 IBCs and 2,351 non-IBCs. The analysis focused on 756 different genes present in at least one targeted NGS panel used across the represented series. For the first time in literature, all comparisons of DNA copy number and mutational data were adjusted upon both the molecular subtypes and AJCC stage and were corrected for multiple tests. The genomic profiles were heterogeneous in IBC. The TMB was higher in IBCs than in non-IBCs, in agreement with the higher genomic instability and complexity of the disease. Higher TMB, combined with the relatively peculiar immune microenvironment of IBC [9, 10], suggests that immune checkpoint inhibitors warrant investigation in IBC. In agreement, we recently launched the PELICAN trial (NCT03515798), an international multicentric phase II study evaluating pembrolizumab in combination with neoadjuvant chemotherapy in HER2- IBC.

The 10 most frequently genes we found altered in IBCs were *TP53* (63%), *HER2/ERBB2* (30%), *MYC* (27%), *PIK3CA* (21%), *BRCA2* (14%), *CCND1* (13%), *GATA3* (13%), *NOTCH1* (12%), *FGFR1* (11%), and *ARID1A* (10%). We identified 96 genes differentially altered between IBC and non-IBC, including 95 more frequently altered in IBC such as *TP53*, genes involved in DNA repair (*BRCA2*) and NOTCH pathways, and only one (*PIK3CA*) more frequently altered in non-IBC. Genes such as *EZH2* and *SMARCA4*, involved in chromatin remodeling, were also more frequently altered in IBCs, providing a rationale for the evaluation of epigenetic modifiers. Interestingly, 37/96 differential genes were more frequently altered in metastases than in primaries of non-IBC patients, suggesting a possible link of these genes with proclivity to metastasize.

Ninety-seven percent of IBCs displayed at least one AGA. This percentage was higher than in non-IBCs (87%). We analyzed six specific drug classes and functional pathways. In four classes, and even if the percentage of patients with AGAs in IBC did not remain significantly superior (classes of HER/EGFR inhibitors, of other tyrosine kinase receptors inhibitors, and of CDK4/6 inhibitors) or inferior (class of PI3K/AKT/mTOR inhibitors) to that of patients with AGAS in non-IBC in multivariate analysis, the percentages were high in IBC patients. Such observation suggests that, like non-IBC patients, IBC patients may benefit from these inhibitors. Of note, an expression signature associated with sensitivity to palbociclib showed a higher score in IBCs than in non-IBCs.

Two functional pathways gave particularly interesting results. Several genes involved in DNA repair were more frequently altered in IBCs such as *ATM, ATRX, BARD1, BRCA2, ERCC3, MSH2, MSH6, PMS2, and POLE*, confirming recent findings [7]. The percentage of patients with alterations of DNA repair genes was twice as high in IBC as in non-IBC (33% versus 17%). Such deficient DNA repair might contribute to IBC progression, as well as to the high TMB observed. We also found that IBCs showed more frequently (~2.3-fold more) a homologous recombination deficiency (HRD) score, supporting the development of PARP inhibitors in IBC.

Alterations in the NOTCH pathway were almost twice more frequent in IBC (30% vs 17% in non-IBC). *NOTCH1* was the most frequently altered *NOTCH* gene in IBC (12%), and *NOTCH2*, and *NOTCH4* were more frequently altered in IBC compared with non-IBC, as reported [7]. We also found a NOTCH pathway activation score higher in IBC than in non-IBC. Thus, these results support a role for the NOTCH pathway in IBC, previously suggested from pre-clinical models, with for example the ability of a gamma-secretase inhibitor to block NOTCH signaling and to attenuate the stem-like phenotype of IBC cells [11]. NOTCH targeting might be a therapeutic option in IBC.

In conclusion, the genomic landscape of IBC is different from that of non-IBC, independently from molecular subtypes and stage. The high percentage of patients with AGA suggests that precision medicine is a *bona fide* option in this aggressive disease, notably with drugs targeting immune system, DNA repair, NOTCH signaling, and CDK4/6. Functional and clinical validation is warranted and clinical trials are ongoing. But clearly, analysis of larger series of IBC samples with larger-scale NGS (Whole-Exome Sequencing, Whole-Genome Sequencing), RNA-Seq, epigenomics and other technologies is needed, though collaborative studies we launched. Such analyses will allow better assessment of structural variations, mutational signatures, clonality and epigenetic alterations and could reveal the etiology of this mysterious and devastating disease.
